# Strengthening of Wood-like Materials via Densification and Nanoparticle Intercalation

**DOI:** 10.3390/nano10030478

**Published:** 2020-03-06

**Authors:** David Novel, Simone Ghio, Andrea Gaiardo, Antonino Picciotto, Vincenzo Guidi, Giorgio Speranza, Maurizio Boscardin, Pierluigi Bellutti, Nicola M. Pugno

**Affiliations:** 1 Laboratory of Bio-Inspired, Bionic, Nano, Meta Materials & Mechanics, Department of Civil, Environmental and Mechanical Engineering, University of Trento, via Mesiano 77, I-38123 Trento, Italy; novel@fbk.eu (D.N.); simoneghio1@gmail.com (S.G.); 2 Centre for Materials and Microsystems, Fondazione Bruno Kessler, via Sommarive 18, I-38123 Trento, Italy; gaiardo@fbk.eu (A.G.); picciotto@fbk.eu (A.P.); speranza@fbk.eu (G.S.); boscardi@fbk.eu (M.B.); bellutti@fbk.eu (P.B.); 3 Department of Physics and Earth Science, University of Ferrara, via Saragat 1/c, I-44122 Ferrara, Italy; guidi@fe.infn.it; 4 Istituto di Fotonica e Nanotecnologie & Consiglio Nazionale delle Ricerche IFN—CNR, via alla Cascata 56/C Povo, I-38123 Trento, Italy; 5 Department of Industrial Engineering, University of Trento, via Sommarive 9, I-38123 Trento, Italy; 6 School of Engineering and Materials Science, Queen Mary University of London, Mile End Road, London E1 4NS, UK; 7 Ket Labs, Edoardo Amaldi Foundation, via del Politecnico snc, I-00133 Rome, Italy

**Keywords:** cellulose-based nanocomposites, nanoparticles, densification, alkaline treatment, giant reed, structural modifications of natural materials

## Abstract

Recently, several chemical and physical treatments were developed to improve different properties of wood. Such treatments are applicable to many types of cellulose-based materials. Densification leads the group in terms of mechanical results and comprises a chemical treatment followed by a thermo-compression stage. First, chemicals selectively etch the matrix of lignin and hemicellulose. Then, thermo-compression increases the packing density of cellulose microfibrils boosting mechanical performance. In this paper, in comparison with the state-of-the-art for wood treatments we introduce an additional nano-reinforcemeent on densified giant reed to further improve the mechanical performance. The modified nanocomposite materials are stiffer, stronger, tougher and show higher fire resistance. After the addition of nanoparticles, no relevant structural modification is induced as they are located in the gaps between cellulose microfibrils. Their peculiar positioning could increase the interfacial adhesion energy and improve the stress transfer between cellulose microfibrils. The presented process stands as a viable solution to introduce nanoparticles as new functionalities into cellulose-based natural materials.

## 1. Introduction

Nowadays, research on natural materials and their technological optimization play a fundamental role in sustainable socio-economic development [1,2]. In particular, cellulose-based materials such as wood, bamboo, reed, and natural fibers have attracted enormous attention [3,4,5,6,7], given their high versatility suitable for a variety of applications including renewable energy source, fuel, comfort, furniture, construction, and recyclable packaging [4,8,9,10]. Moreover, chemically treated wood can be turned into a transparent and conductive substrate for solar cells applications, flexible transistors, and electronics display such as liquid-crystal display (LCD) and organic light-emitting diode (OLED) [11,12]. In addition to the aforementioned applications, the most extensive utilization of timber since ancient times has been as a construction material, and it is still nowadays preferred over more modern building materials in a lot of instances due to a better life-cycle assessment, greater energy efficiency, lower costs, and near-to-zero waste by-product [13].

Wood, bamboo, and reed represent valid alternatives to steel and concrete not exclusively for socio-economic reasons but also for their structural qualities [7,14,15,16,17]. Through a combination of high strength and lightness, wood-like materials are strong competitors to modern construction materials [13]. Natural hollow materials, such as bamboo and reed, have superior tensile and flexural strength than wood [7,16,17]. Regarding these distinctive features, the exploitation of the intrinsically superior strength of hollow materials in the creation of larger and complex artefacts can generate a new strong competitor in the roster of construction materials. Nonetheless, the bare mechanical properties of bamboo are superior to those of giant reed and reed is considered to be a highly invasive and unwanted species [16,18,19,20].

The first historical records of the use of giant reed (arundo donax) dates back to Ancient Greece [21]. Nowadays, limited applications use reed, i.e., biofuel [22], industrial cellulose source [21], reed wattle for structural purposes, biomimetic, and technical textiles applications [6]. Due to its high biomass productivity [22], it can be exploited as a sustainable fast-growing and low-cost material [21].

The strength of arundo donax relies on its structural composition comprising cellulose fibers and hemicellulose embedded in a lignin matrix [19]. The cross-section analysis of reed culm showed a structure with a homogeneous density of fibers, which is increased in the cortical region being more rigid [19], showing mechanical properties superior of most type of woods [17]. Giant reed has developed, similarly to bamboo [5], an optimal stem structure, which is reinforced with internodes to overcome the weakness due to its hollow and slender conformation (i.e., Euler buckling) [20]. In fact, the internodes allow the giant reed to withstand high compressive and flexural loads, which would be impossible without their presence.

This work focuses on a novel chemical and structural modification to turn giant reed into an advanced material with extremely improved mechanical properties and higher stability under challenging environmental conditions. Giant reed specimens were treated in an alkaline environment to decrease the concentration of lignin and enable densification [23,24]. The removal of lignin and thermo-compression allowed to achieve a denser material with high structural performance [24]. Porosities occur because of the delignification process. We propose to exploit even these tiny spaces, filling them up with nanoparticles to make the material tougher.

Notwithstanding, several authors investigated the effect of inorganic nanostructured materials on wood or bamboo [25,26,27,28]. In this work we propose to tailor the effects of densification by introducing selected nanostructured materials in the structure of giant reed, i.e., silicon carbide (SiC) and graphene oxide (GO). On the one hand, SiC is a well-known material for its unique mechanical strength, which was exploited to improve the mechanical properties of metals [29], polymers composites [30], and cellulose-based materials [31]. On the other hand, GO flakes were chosen for its 2D structure with high aspect ratio exposing a high concentration of hydrogen bonds [32] to the cellulose/lignin units present in the reed as GO flakes proved to effectively enhance mechanical performance in cellulose-based materials [33]. The final yield of this process can further improve the strength and toughness compared to the same densified reed. The analyses carried out highlighted a significant increase in the mechanical properties of densified samples intercalated with nanoparticles, especially regarding stiffness, strength, and toughness. The addition of nanoparticles in the densified reed resulted also in a reduced water uptake by the reed, which decreases its biodeterioration [16,34] and thus guarantees a longer lifespan. Furthermore, the densified and nanoparticles intercalation treatments strongly increased the fire-retarding properties of the native reed and the “thermo-indentation” resistance, measured by using a custom-developed technique.

## 2. Materials and Methods

Three years old giant reed, i.e., arundo donax, was used for this study. A mixture of sodium hydroxide (Sigma-Aldrich), sodium sulfite (>98%, Sigma-Aldrich, St. Louis, MO, USA), and deionized (DI) water was used for the partial removal of lignin from the reed. After the lignin etching, reed specimens were immersed in silicon carbide (3C–SiC, purity >99%, Tec Star, Castelfranco Emilia, Italy) or graphene oxide (GO, water dispersion 4 mg/mL, monolayer content >95%, Graphenea, San Sebastian, Spain). A Bench Press (Pneumtic Table Top Press 2.5 Tons by Gibitre Instruments, Bergamo, Italy) was used to thermo-compress samples.

All the specimens were extracted from the culm of giant reed. Their orientation was parallel to sclerenchyma fibers, and they were mechanically polished to a regular geometry (10 × 2 × 0.5 cm^3^). The cross-section of samples is perpendicular to the fibers’ direction, while the lateral side is parallel to them. Then, the specimens were immersed in a re-fluxed boiling water solution containing NaOH (2.5 M) and Na_2_SO_3_ (0.4 M) to partially remove lignin.

The initial concentration of lignin for the arundo donax samples can be inferred from literature, when the age of the giant grass is known. In our case, the starting content of Lignin is about 22% [35]. The exposition time in alkaline solution was chosen according to the results achieved by Song et al., which showed that a decrease of about 50% in lignin content delivers the best mechanical properties [24], we conclude that the final concentration of lignin in our experiment is about 11%. Chemicals were removed from etched specimens by thoroughly washing samples in several boiling baths of deionized water, followed by washings with running deionized water. Chemically etched samples after delignification were dried overnight at 30 °C.

Afterwards, three sets of samples were prepared and each of them was subjected to a different treatment. The first reference set (labelled: D) was immersed in water, the second (D + SiC) was intercalated with SiC nanoparticles, and the third (D + GO) with GO (graphene oxide) flakes. To this aim, giant reed specimens were immersed in SiC and GO aqueous solutions (both at a concentration of 1 mg/mL). Then, vacuum was applied to remove trapped air inside samples and the solutions were vigorously stirred for 24 h at room temperature. An autoclave treatment of 3 h at 5 bar pressure followed. Last, while being still wet, each set was hot-pressed at 100 °C and 5 MPa of pressure for 3 h. Properties of these samples are compared with two other sets: Natural reed (labelled R) and natural reed subjected only to thermo-compression (TC) at 100 °C under 5 MPa pressure for 3 h, an intermediate treatment useful as a comparison as it is already implemented in the industry [7].

Mechanical properties were measured with a displacement controlled, electromechanical testing machine from Messphysik Materials Testing (MIDI 10). Tensile testing was carried out five times for each of the five sets of samples. The speed of each test was computed to get the same strain rate in both the tensile tests and the lower fibers of samples under flexural loading. The Young’s elastic modulus was calculated for all samples in the same deformation range, from 0.025% to 0.075%, which displays a linear elastic region for all samples. Stress-strain curves are reported until fracture.

Flexural tests were carried out with a three-point bending setup. Flexural tests were carried out three times for each of the five sets of samples. The span length between the two support cylinders was 19.2 mm, this distance was increased to 50 mm for natural bamboo to fulfill the geometry requirements of the ASTM Standard D790-17. The cross-head speed was computed to obtain a suggested 0.01 mm/mm/min of strain rate on the outer fiber of the flexural samples. Flexural stress-strain curves are reported until the maximum load.

Microstructural studies were performed with a field emission scanning electron microscope (JSM-7401F, JEOL Ltd., Tokyo, Japan) and profilometer technique. A stylus profilometer (P6, KLA Corporation, Milpitas, CA, USA) was used to analyze the cross-section of the five sets of samples and to reconstruct their 3D surface by merging line scans having a distance of 2 µm.

Roughness was calculated with root mean square (RMS) on a wide area. The morphology and chemical composition of specimens were analyzed by SEM and energy dispersive X-ray spectroscopy (EDX spectroscopy) techniques, by means of a SEM.

Burning tests were performed using a modified Taghiyari method [28] and a thermographic camera. The classical Taghiyari method has been modified to better fit the thermographic inspection of the process, and a Bunsen type burner was used to generate the free flame. The wood samples were placed perpendicularly to the floor, at 90° with respect to the flame direction, with the width facing the camera and the thickness directly above the flame. The normalized burned volume was computed as follows:

(1) $$V_{\rho }\left(t\right)=\frac{A\cdot d\left(t\right)}{\rho }$$

where *V_ρ_(t)* is the normalized burned volume, A and *ρ* are the cross-section and density of the sample, and *d(t)* is the flame advancement and was computed considering the area having a sample temperature higher than 360 °C. In order to be able to cross compare the calorific value of samples with different microstructure, the burned volume was normalized by the density since the calorific value is related to sample mass, hence to the density if a constant volume is considered.

Thermo-indentation tests were performed using a hot conical tip indenter (17.7° apex angle) at 400 °C. The indenter was placed perpendicular to the lateral surface of the surface with an indentation force of 0.65 N for 5, 10, and 15 s. The burned area was calculated from the images of samples’ surfaces.

Accelerated humidity absorption tests were performed in a climatic chamber with controlled humidity 97 ± 1% rH. The values of humidity absorption are reported in a mass increase from the dried state. At every sampling time five samples—one per set—were extracted from the chamber, transferred in a closed vial, and weighed.

The chemical composition of the virgin and treated samples was examined also by X-ray photoelectron spectroscopy (XPS) (Axis DLD Ultra, Kratos Analytical Ltd., Manchester, UK). The analysis consists of the acquisition of a wide spectrum at a pass energy of 160 eV to detect all the chemical elements constituent of the sample surface. Then, core lines of interest were acquired at a higher energy resolution using a pass energy of 20 eV. Since samples are nonconductive, charge compensation was needed. Optimal conditions of compensation were obtained minimizing the full width at half maximum of the core line peaks and maximizing their intensity. This leads to an energy resolution of ~0.3 eV. Finally, data reduction was performed using a software made in-house based on the R platform (https://www.r-project.org). For each core line, a linear background subtraction and Gaussian components were used for peak fitting.

## 3. Results and Discussion

**Figure 1:**
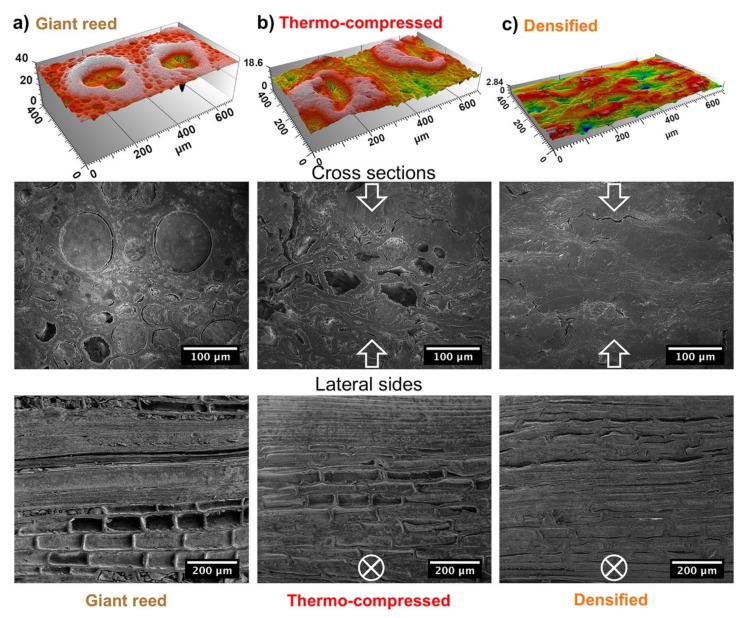
The first row of images shows the cross-section morphologies of (**a**) R, (**b**) TC, (**c**) D samples obtained by profilometry analysis. The second and third rows show SEM images of the cross sections and the lateral sides of (**a**) R, (**b**) TC, (**c**) D. White arrows in SEM images show the direction of applied pressure in the thermo-compression treatment.

Sample morphologies were analyzed by SEM and scanning profilometry to reveal the effects induced in the internal structure of giant reed by the treatments applied. To this aim, specimens were embedded in epoxy resin and polished to obtain a macroscopically flat surface for both the cross-section and the lateral side. The profilometry analysis on the lateral face (Figure 1) is very useful to evaluate the packing density of the internal parenchyma structure. These structures tend to collapse under the effect of the external pressure. The collapse is partial for thermo-compressed (TC) reed (Figure 1b) and it leads to a reduction in the lateral size of the cellular structure ranging from 65 to 46 µm. Conversely, densified (D) giant reed (Figure 1c) show an almost complete collapse in parenchyma cells and their lateral size shrinks down to ~20 µm. Similarly, the effects of densification are also reflected on the roughness of the cross-section surface. Compressed samples are increasingly denser and more compact (Figure 1b,c) than untreated reed (Figure 1a). The roughness, quantified as the root mean square of the height, is reduced by 7-fold, from 2.68 to 0.36 µm after densification. This reduction is mostly due to partial removal of the lignin that accounts for the soft part [36,37] of the untreated reed matrix while, in the densified samples, stiff cellulose microfibrils (CMFs) become the predominant component [36,37]. As already noted for the collapse of parenchyma structures, thermo-compression represents a partial treatment. Indeed, the roughness is slightly reduced, from 2.68 to 2.44 µm.

As just discussed, the different treatments have an effect on the structure of materials, and they involve a density change. The different mass densities are 0.468 ± 0.015, 0.804 ± 0.086, 1.259 ± 0.010, 1.265 ± 0.035, 1.279 ± 0.031 g/cm^3^ for R, TC, D, D + GO and D + SiC specimens, respectively. It is worthy of attention that the density of D sample is still lower than that of cellulose [38], which ideally corresponds to a complete densification and lignin removal. In fact, lignin is not completely removed in D and there are still some voids after densification. Moreover, the densification treatment has proven to be very effective in the packing of internal structures since the density of D is 8% higher than single reed fibers [6]. The lower packing density of TC compared to D is due to the lack of the lignin removal process, which does not allow to achieve high levels of compaction during thermo-compression.

SEM images (Figure 1) give a clear focus on the various extents of collapse associated with the different treatments that were performed. The first row of SEM images shows the cross sections, the second row shows the lateral side parallel to CMFs. The collapse in the internal structures increases going from left (R) to right (TC, D). We found that the densified reed (Figure 1c) has a far more compact and ordered structure than natural reed, but there is still a presence of some voids between CMFs, which are of approximately several microns long and hundreds of nanometers thick. Both the compact structure and the presence of small voids have a crucial importance for the intercalation of nanoparticles and the formation of intermolecular interactions, such as hydrogen bonding, with the surrounding CMFs.

**Figure 2:**
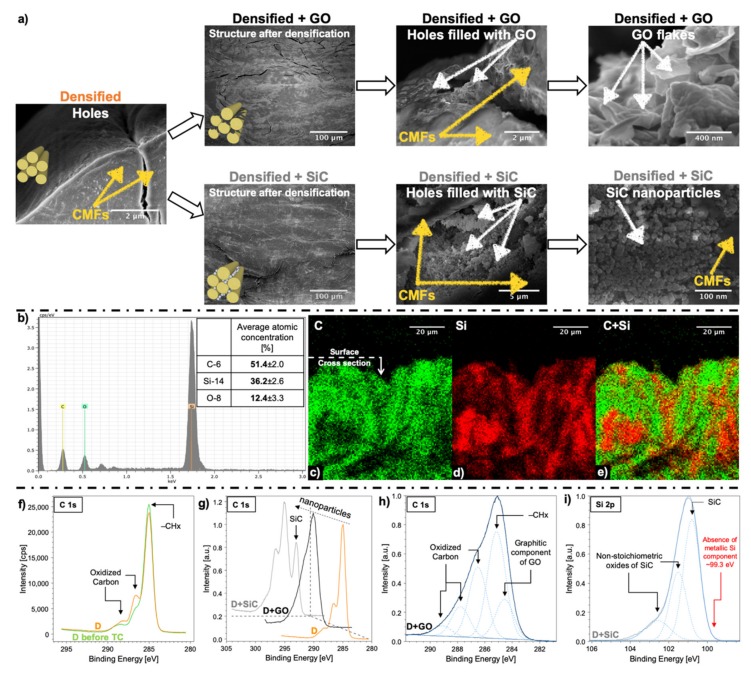
(**a**) SEM images of D, D+GO and D+SiC specimens. The second column displays the D + GO and D + SiC samples after nanoparticles intercalation and densification treatments: There is no evident morphological difference between the two. The third and fourth columns show high magnification SEM images with details of the location of SiC nanoparticles and GO flakes at the interface of cellulose microfibrils (CMFs). GO, SiC, and CMFs are highlighted with arrows. (**b**) EDX spectrum performed at 10 keV. The table reports the average quantitative elemental analysis of carbon (C), silicon (Si), oxygen (O) from three different wide scans sampled next to the surface in SiC-rich regions. Maps of the atomic percentage of the cross-section of D + SiC specimens: (**c**) C, (**d**) Si, (**e**) C + Si. XPS analysis of the (**f**) thermo-compression treatment on lignin etched reed, (**g**) nanoparticles intercalation on densified reed; (**h**) GO flakes, and (**i**) SiC nanoparticles intercalation in the densified reed.

By comparing SEM micrographs of TC and D (Figure 1b,c), it can be clearly seen that TC is less dense than D, while TC is still maintaining a structure similar to R. This resemblance disappears in D, D + GO, and D + SiC (Figure 2a) where the structural features of R cannot be distinguished anymore.

This similarity disappears in D, D + GO, and D + SiC where the structural characteristics of R are no longer observable. Figure 2a shows cross-section SEM images of the D, D + GO, and D + SiC samples, at various magnifications. SiC nanoparticles and GO flakes filled the voids in the D sample (Figure 2a), as initially supposed, forming thin layers at the interface between CMFs.

EDX analysis was carried out to investigate the distribution of silicon in the D + SiC specimens. Both atomic concentration spectra (Figure 2b) and atomic distribution maps (Figure 2c,d,e) have been collected to quantify the percentage of silicon carbide on the specimen surface and its distribution in densified giant reed as a result of SiC intercalation. As it can be seen through EDX signal, the atomic concentration of Si was about 36% at surface (Figure 2b). SiC nanoparticles penetrated the D + SiC sample through the surface and naturally their concentration at surface is higher than in the bulk (Figure 2d). Whereas a rough estimate of the concentration of nanoparticles, calculated from density changes, yields ~0.9 wt% for both D + GO and D + SiC samples.

For the other samples, EDX analysis cannot provide meaningful insights since they are mostly composed of carbon, oxygen, and hydrogen. The sensitivity of EDX technique is not enough to precisely quantify elements with such low atomic numbers. Therefore, the XPS analysis was carried out to have a broader view of the chemical composition, owing to its higher sensitivity to C and O atoms than EDX analysis and its possibility to investigate chemical bonds.

Samples after delignification and after densification were compared to evaluate the effect of the thermo-compression treatment on the material (Figure 2f). Thermo-compression induces a slight conversion of −CHx into epoxy, alkoxy, and carbonyl components (Figure 2f) in the delignified reed. We suspect that alongside delignification, there is a decrease in crystallinity of cellulose (see Figure S1), owing to the alkaline environment [39]. According to the delignification treatment optimized by Song et al. [24], which was here applied on giant reed, it revealed 12% reduction of the cellulose content, while hemicellulose was decreased 4-fold.

Figure 2g shows different bonds in densified reed after introducing the GO flakes and SiC nanoparticles. D + GO spectrum shows a graphitic shoulder at binding energies lower than (−CH_x_) bonds. As well as, an increase in all the oxidized carbon components (C–O, C=O and O=C–O) can be assumed from Figure 2h. By adding SiC nanoparticles, a new peak arises at ~283 eV, as shown in Figure 2i for the Si 2p line. Presence of GO flakes, when compared to D spectrum (Figure 2g), generates a significant increment of all the oxidized carbon components (Figure 2h) in the overall structure of densified reed. There is also the rise of a graphitic shoulder ~284.6 (Figure 2h) owing to the molecular structure of GO flakes. The Si^0^ component, which should fall at ~99.3 eV (Figure 2i), is absent and the narrow peak of SiC (Figure 2i) demonstrates that SiC nanoparticles are crystalline. In addition, some native oxides are formed on the surface of SiC nanoparticles, as denoted by the presence of the nonstoichiometric oxide components in the Si 2p XPS spectrum (Figure 2i).

**Figure 3:**
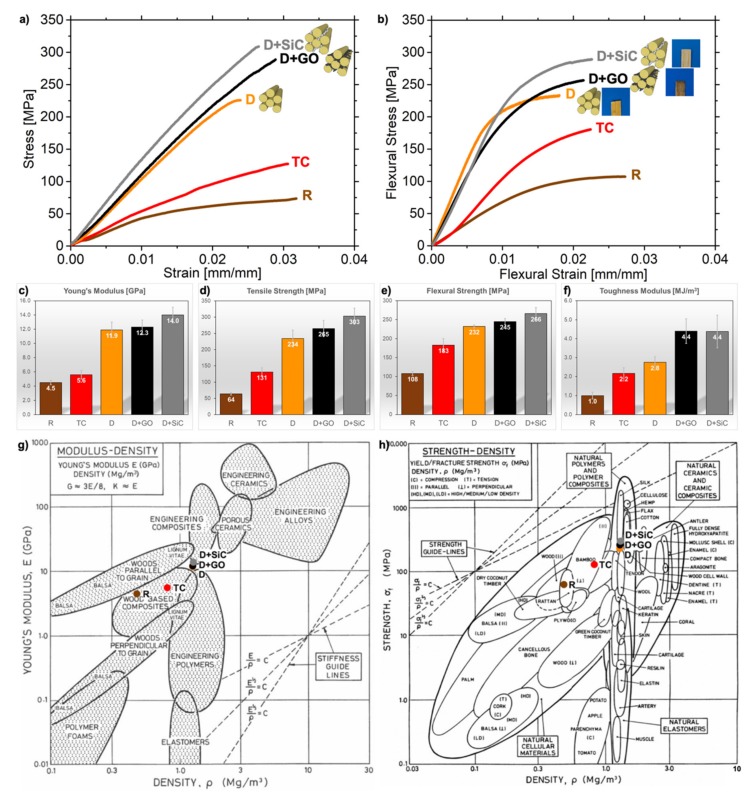
Average representative (**a**) stress-strain and (**b**) flexural stress-strain curves. Simplified representation of CMFs with GO flakes and SiC nanoparticles is given for D, D + GO, D + SiC. After the addition of nanoparticles, samples’ color change as shown (D + GO veers towards black while D + SiC veers towards gray). Histograms summarizing the properties of R, TC, D, D + GO, and D + SiC samples: (**c**) Young’s modulus, (**d**) tensile strength, (**e**) flexural strength, and (**f**) toughness modulus (area under the tensile stress-strain curve). Error bars represent the standard deviation. Material properties charts show the five materials of this work compared to common engineering materials: (**g**) Young’s modulus vs. density chart and (**h**) strength vs. density chart - adapted from [17] with permission. In the Ashby charts, the final densified products sit above the strength and stiffness guidelines traced using reed as the reference point.

The evaluation of treatments’ effect on the mechanical properties are analyzed in this section. The stress-strain curves of the uniaxial tension test are reported in Figure 3a,b. It is worthy of attention that flexural properties follow the same trend of tensile tests, as shown in Figure 3.

All samples exhibit a brittle behavior. D, D + GO, D + SiC display a mostly linear loading curve, typical of natural fibers with high cellulose content [40]. Non chemically-treated samples, which have higher lignin concentration, show a nonlinear loading curve at low strains linked to the progressive loading of cellulose fibers through the deformation of cell walls [6] followed by a viscoelastic response, whose contribution mainly depends on amorphous regions (lignin, hemicelluloses, pectins) [6,41].

From the stress-strain curves (Figure 3a) we computed a Young’s modulus for the giant reed of 4.5 GPa (Figure 3c), which agrees with the measurements of Speck et al. [42]. After densification, stiffness rises to 11.9 ± 1.1 GPa and likewise the tensile and flexural strengths results in a drastic improvement of 3.66 times for the tensile strength (from 64 ± 4 MPa in R to 234 ± 26 MPa in D, Figure 3d) and 2.15 times for flexural strength (from 108 ± 5 to 232 ± 4 MPa, Figure 3e). Hence, the obtained performance of densified reed is superior to regenerated cellulose films [43] and very similar to both those of isolated reed fibers [6] and cellulose nanopaper [11]. Moreover, the properties achieved through densification are superior to many types of bamboo [7,16,44], resulting in a highly competitive construction material (Figure 3g,h).

Introducing nanomaterials grants an additional boost to the performance of densified reed. Indeed, the Young’s modulus rises to 12.3 ± 1.0 for D + GO and 14.0 ± 1.1 GPa for D + SiC (Figure 3c), which are higher than most engineering polymers (Figure 3g). Therefore, these materials moved from the wood’s group in the Ashby chart (Figure 3g) to engineering composites. Accordingly, both tensile strength (+ 13% for GO and + 30% for SiC) and flexural strength (+ 5% for GO and + 15% for SiC) are improved (Figure 3d,e). These enhancements in mechanical properties are caused by the addition of nanomaterials, which improves the interfacial adhesion between CMFs. Densified nanocomposites fall into the density category of biological materials designed to work under tension, such as tendons, but they show higher strength. As expected, the upper limit in terms of both density and strength for the nanocomposites is cellulose [17,45], which is the strongest structural component of reed. However, their strength is superior to wood and plywood (Figure 3h).

Nanoparticles improved the deformation capabilities in bending (+ 10% for D + GO and + 20% for D + SiC, see Figure S2). Composite wood-like structures have a predominant deformation by shear, which is concentrated in the soft matrix [46], rather than on the stiffer cellulose fibers. It is clear that, with a partial removal of the lignin and hemicellulose matrix, the deformation capabilities of reed are reduced [47]. Indeed, densified reed exhibits lowered strain to failure in both tensile and flexural tests (see Figure S2). However, the intercalation of nanoparticles generates a remarkable increase of the materials’ toughness modulus (Figure 3f). It goes from 2.8 MJ/m^3^ after densification, which is the current state-of-the-art process, to 4.4 MJ/m^3^ for D + GO and D + SiC.

To further compare the mechanical properties of each material, specific toughness modulus was taken into account. It is calculated by dividing the area under stress strain curves (i.e., toughness modulus) by the density. As a result of the variations in packing density introduced by the treatments, specific toughness modulus is suitable to evaluate the intrinsic improvements in toughening efficiency rather than toughness modulus. This is due to the increase in the packing density induced by compression, owing to a toughness modulus increase while the specific toughness modulus remains the same.

No marked difference in specific toughness modulus (see Figure S2) surfaces in the three samples of R, TC and D. Instead, the intercalation of nanoparticles acts as a further reinforcement on top of the previous densification treatments as it generates an improvement in the specific toughness moduli of +59% for D + GO and +56% for D + SiC (Figure S2). The toughening could be caused by the location of nanoparticles at the interface between CMFs (Figure 2a). Indeed, both SiC nanoparticles and GO flakes were intercalated into the pores of giant reed, which were opened through chemical treatment and then closed by thermo-compression (Figure 1, Figure 2a). It can be interpreted in terms of better interfacial adhesion or increased in the chemical cross-linking at the interface driven by the additional hydrogen bonds available from the GO structure [48,49] and the nonstoichiometric oxides shell (Figure 2i) on SiC nanostructures [50,51]. Not to mention that the small amount of Ca impurities, which was found in all the samples (see Figure S1), was reported to have a beneficial effect on the chemical cross-linking between GO and cellulose [52], thus effectively enhancing the stiffness and the strength between GO layers [53]. It was also demonstrated that the densification process on wood at moderately high temperatures can lead to the formation of new hydrogen bonds between CMFs [12,24], which is here favored by a high degree nanofibers’ alignment. The higher level of oxidation emerging from XPS analysis (Figure 2f) and the formation of new hydrogen bonds leads to a highly densified material that is stronger than the same sample compressed at room temperature.

As a result of the intercalation of nanoparticles, we estimated an average improvement of the interfacial fracture energy between CMFs that is 1.28 ± 0.33 for D + GO and 1.47 ± 0.36 for D + SiC, both compared to the D sample (see Supplementary). The fracture energy was calculated according to an analytical model [54] and it is proportional to σ^2^/(E*R), where *σ* is the tensile strength, *E* is the Young’s modulus of the material, and *R* is the radius of the CMFs.

**Figure 4:**
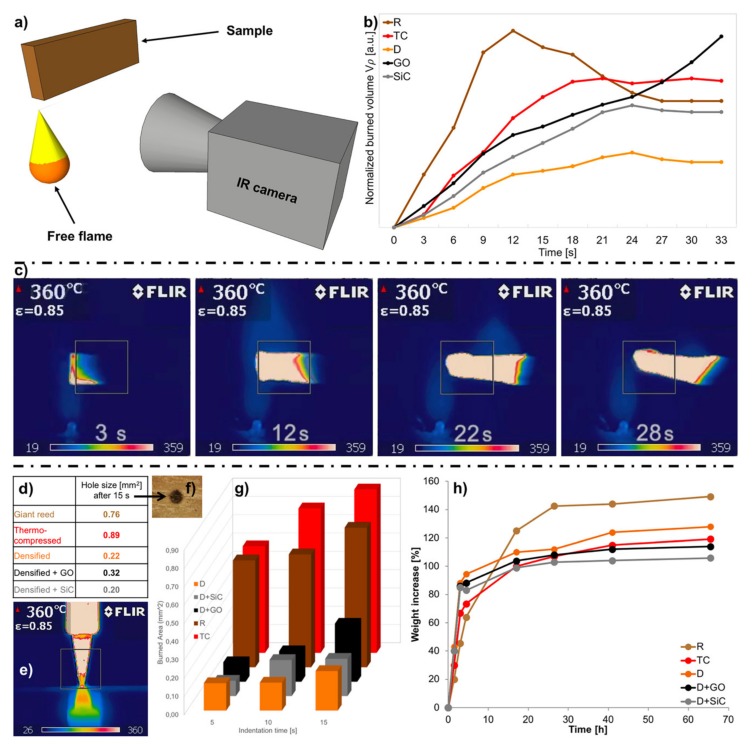
(**a**) Schematic representation of the burning test setup. (**b**) Burning test results of all specimen types. (**c**) Time evolution of thermal imaging data from D + GO sample (complete videos are available in SI). (**d**) Table showing the burned area after the 15 s of thermo-indentation (**e**) thermographic image of the indenter tip (**f**) burned area results of all specimens when subjected to 5, 10, and 15 s of thermo-indentation (**g**) burned area vs. indentation time. (**h**) Accelerated humidity absorption tests carried out on R, TC, D, D + GO, and D + SiC specimens. The graph represents the weight increase of samples vs. exposition time at constant 97% of relative humidity.

Two different tests were performed to evaluate the burning resistance of the samples: A free flame burning test and a thermo-indentation. A modified Taghiyari method [28] was used to perform the free flame burning test (Figure 4a).

Figure 4b shows the comparison between all the samples. The normalized burned volume is calculated as reported in the Methods section. During the first half of the test the reed burns faster than its compressed and densified counterparts, then it auto-extinguishes itself faster than the TC sample. Regarding the overall resistance to free flame, the densification treatment yields the best results as it performs almost twice better than all other samples.

All samples showed fire extinguishing properties (Figure 4b). The only exception is D + GO. After about 20 s, where all curves start to slow down, the specific burned volume of D + GO accelerates again. This effect cannot be entirely attributed to the high thermal conductivity of GO. In fact, differential scanning calorimetry (DSC) and thermogravimetric analysis TGA curves of GO^48^ show a strong exothermic reaction at about 200 °C, the energy release associated with it can drive flame propagation and accelerate the burning of the sample depicted by the black line in Figure 4b. Furthermore, the Na impurities that remain in the structure after the delignification process (see XPS spectra in Figure S1) interact with GO flakes to accelerate the burning mechanism [55,56]. In general, the introduction of nanoparticles seems to reduce the flame retarding properties that the D shows compared to the R samples.

Ignition of wood can result from free flame or contact with a hot body. An ad-hoc thermo-indentation test was designed to quantify the resistance of each sample to burn in contact with a hot body.

The thermo-indentation was performed using a hot tip perpendicular to the surface (Figure 4e). The burned area from the tip was used to evaluate the data (Figure 4f). These measurements depend both on the specific burning features of the samples and on the elastic modulus of the surface of the indented sample. The thermo-indentation performance can be explained cross comparing the data of the specific burning test with the elastic modulus. In fact, both R and TC have higher indentation area and TC shows a higher burned area compared to R (Figure 4g). Furthermore, the densified and nanocomposite reed have a lower burned area than both TC and R, due to higher burning resistance and higher Young’s modulus of D, D+GO and D+SiC (Figure 4g and Figure 3c), which increase thermo-indentation performance. Moreover, also during the thermo-indentation of the densified sample with GO nanoparticles, the burned area saw an upright increment from 10 to 15 s that did not arise in the other samples (Figure 4g). In conclusion, both graphs of free flame and thermo-indentation show similar trends, apart from D + GO in the burning test due to the aforementioned effect (Figure 4b,c). All densified samples showed superior resistance to thermo-indentation compared to R or TC, with D + SiC having the best resistance of all for longer times (Figure 4g).

The humidity absorption characteristics of the reed samples were calculated by the percentage of weight gain at different times. Interestingly, the humidity absorption kinetics differs with the treatments applied (Figure 4h). The two samples that contain all the lignin (R, TC) have a slower initial growth but have a high final steady state. The presence of lignin has a crucial impact on the water absorption since it is a cross-linked phenolic polymer with few terminal hydroxyl groups and low water solubility [39]. Therefore, when lignin is removed (D, D + GO, D + SiC), the water gain is faster but has a lower final asymptote. This finding is correlated with the higher packing density of cellulose microfibrils in densified samples and with the increased capillarity effect that is induced by the chemical treatment [57].

The asymptotic values, which samples approximately reach after 40 h, have a maximum value of + 150% for R that has the highest moisture absorption whereas they are significantly lower when nanoparticles are added: 114% for D + GO and 106% for D + SiC. The latter ranks as the lowest absorbing specimen (Figure 4h).

In real-life scenarios the overall absorbed water is the key factor for the survival of wood and reed in critical environments. Less absorbed water will result in lower fungus development [34] and lower degradation in mechanical properties [16]. Thus, densified nanocomposite specimens will perform better under these conditions.

## 4. Conclusions

In this work, we presented an innovative and simple nano-reinforcing treatment for wood-like specimens that involves densification of cellulose fibrils combined with intercalation of GO and SiC nanoparticles. In particular, the process was here tested to improve the mechanical properties of an invasive plant, giant reed, into a stronger and tougher engineering composite and can be applied to other cellulose-based materials such as wood and bamboo.

Morphological and structural characterizations, performed by using profilometer, SEM, EDX, and XPS techniques, highlighted that the treatment performed intercalated the nanoparticles inside the densified giant reed. Although, nanoparticles led to no apparent influence on the structure of the densified reed, their presence and location at the interface of CMFs resulted in a better interfacial adhesion and overall improvement in mechanical performance, both for tensile and flexural properties. Indeed, the mechanical properties of the nanocomposite increase with respect to both the untreated and densified reed. The nanocomposite modified giant reed has a 1/15 of the elastic modulus and more than 1/2 of the strength of mild steel while having just 1/7 of its density. Further analysis highlighted that the nanoparticle intercalation in the densified reed enhanced the specific toughness modulus while this does not occur with the densification treatment alone. This result is due to the role played by nanoparticles during the relative sliding between cellulose microfibrils inside the material. A mechanical effect coupled with an increase in the chemical interaction could be at the root of this phenomenon. Indeed, the densification treatment involves the formation of a high number of hydrogen bonds between CMFs. The amount of these bonds could further increase by adding nanoparticles in the voids of the densified structure, especially with GO, which is rich in hydroxyl and carbonyl groups in its surface. Additionally, an increase in adhesion energy, that was estimated to be higher for SiC nanoparticles than GO, could induce an increase stress transfer between cellulose microfibrils. Further investigations were performed to evaluate thermo-indentation and burning response of the materials. SiC and GO-densified giant reed have strongly increased fire-retarding and thermo-indentation resistances compared to the native reed. Moreover, the addition of nanoparticles in the densified reed resulted in a reduced water uptake, which can decrease its bio-deterioration and thus guarantee a longer lifespan when applied as a structural material. The versatility of this innovative treatment can be exploited to improve the mechanical properties of any kind wood-like structure. In conclusion, the nano-reinforcing densification treatment proposed in this work sheds a new insight into the possible enhancements of timber properties.

## Supplementary Materials

The following are available online at https://www.mdpi.com/2079-4991/10/3/478/s1, Figure S1: XPS, Figure S2: Mechanical properties, Figure S3: Interfacial fracture energy, Table S1: Interfacial fracture energy improvements, Video S1: Burning tests.
