# Supplementary material for: Strengthening of Wood-like Materials via Densification and Nanoparticle Intercalation
Source: Nanomaterials (Basel). 2020 Mar 6;10(3):478. doi: 10.3390/nano10030478 (PMC7153609; doi:10.3390/nano10030478)
Supplement: Supplementary file 1 [file nanomaterials-10-00478-s001.zip › nanomaterials-709726-SP/nanomaterials-709726-SP.pdf]

nanomaterials

SUPPLEMENTARY MATERIALS

Strengthening of Wood-like Materials via Densification and Nanoparticle Intercalation

David Novel [1,2], Simone Ghio [1,2], Andrea Gaiardo [2,3], Antonino Picciotto [2], Vincenzo Guidi [3], Giorgio Speranza [2,4,5], Maurizio Boscardin [2], Pierluigi Bellutti [2], Nicola M. Pugno[1,6,7,*]

1 Laboratory of Bio-Inspired, Bionic, Nano, Meta Materials & Mechanics, Department of Civil, Environmental and Mechanical Engineering, University of Trento, Via Mesiano 77, I-38123, Trento, Italy
2 Centre for Materials and Microsystems, Fondazione Bruno Kessler, via Sommarive 18, I-38123 Trento, Italy
3 Department of Physics and Earth Science, University of Ferrara, Via Saragat 1/c, I-44122 Ferrara, Italy
4 Istituto di Fotonica e Nanotecnologie & Consiglio Nazionale delle Ricerche IFN - CNR, via alla Cascata 56/C Povo, I-38123 Trento, Italy
5 Department of Industrial Engineering, University of Trento, v. Sommarive 9, I-38123 Trento, Italy
6 School of Engineering and Materials Science, Queen Mary University of London, Mile End Road, London, E1 4NS, United Kingdom
7 Ket Labs, Edoardo Amaldi Foundation, Via del Politecnico snc, I-00133, Rome, Italy

Note 1 - XPS:

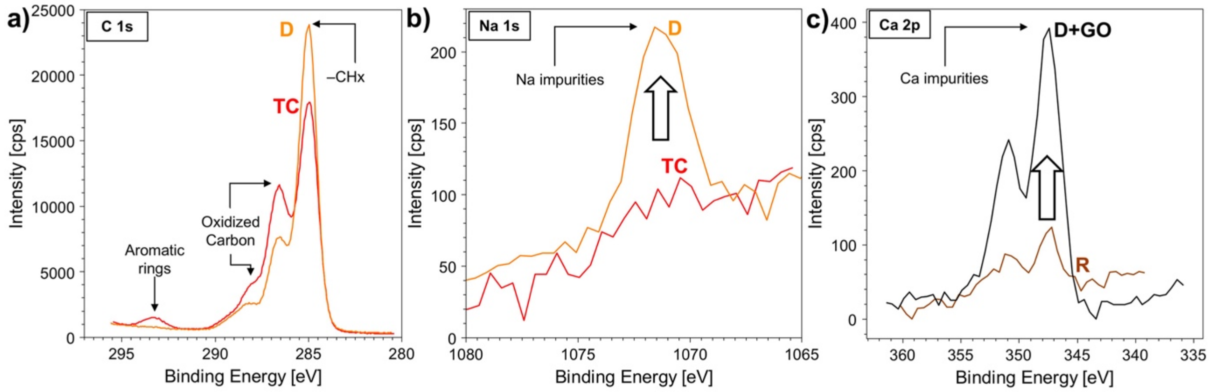

Figure S1. a) The difference in XPS spectra between TC and D shows the effects of delignification. TC and D samples differs only for the delignification process. b) presence of Na impurities. They are increased after the chemical etching c) presence of Ca impurities. They are increased from <0.1% to ~0.5% after the chemical etching. Ca impurities have an important effect on the D+GO as they interact with GO during burning.

The binding energy that is associated with the structure at ~294.3 eV in the C1s spectrum of "TC" in Fig. S1 has energies that are too high to justify the bonding of carbon with any element. It could instead be explained with a shake-up structure that is formed due to the presence of aromatic rings. This structure appears only in R and TC, and it disappears in all the other specimens, which were subject to delignification process. Thus, it could arise from the aromatic rings in the molecular structure of lignin [1]. As Fig. S1 shows, delignification took place going from TC to D and this corresponds with the decrease of the structure at ~294.3 eV. However, the decrease of this feature is not enough to explain the increase of the component associated with –CH_x at 285.5 eV [2] to the detriment of the oxidized carbon that occurs as a result of the chemical delignification process.

A possible concurrent phenomenon is the degradation of hemicellulose and cellulose [3]. Indeed, alkaline treatments on wood-like materials remove part of the lignin and partially depolymerize hemicellulose and cellulose [4]. The subsequent degradation is turned into an extraction of the

different sugars forming hemicellulose or the glucose from cellulose. In particular, in solutions with increased alkalinity, the extraction of glucose from the depolymerization of cellulose in natural fibers is increased [5]. This extraction can reduce the overall amount of oxidized carbons.

Note 2 - Mechanical properties:

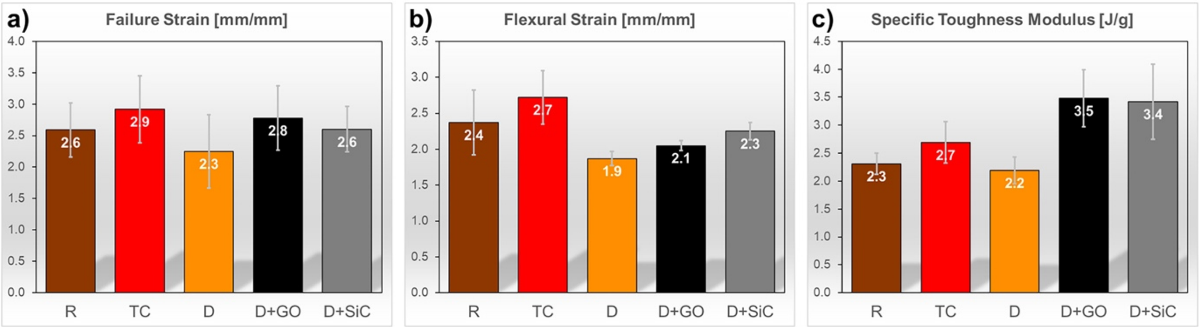

Figure S2. Histograms summarizing the mechanical properties of R, TC, D, D+GO and D+SiC samples: a) failure strain, b) flexural strain and c) specific toughness modulus, which was derived from the toughness modulus as described in the article. Error bars represent the standard deviation.

Note 3 - Interfacial fracture energy:

As for wood-like structures, the mechanical performance of densified and composite reed is dominated by the shear load transfer between cellulose microfibrils [6].

Thus, failure mechanics is prevalently governed by the shear failure at interfibrillar interface. Griffith linear elastic fracture mechanics have proven to be suitable describing the failure of adhesive joints subjected to complex loading conditions [7, 8]. It was suggested [9] that there is a correlation between the load necessary for the crack to propagate and the fracture energy of the interface joint. This insight could result in a better understanding of the adhesion in our nanocomposite materials. The microscale interactions between nanoparticles and cellulose microfibrils can be treated within a precise geometrical configuration that is modelled to estimate the increase in toughness reported after the introduction of nanoparticles. We can imagine the interface as an adhesive joint that unbinds when crack propagates. Fig. S2 shows a hypothetical loading setup for a group of fibrils in mutual contact. An isotropic linear elastic behavior is assumed for the materials involved, i.e. CMFs and interface materials. If the adhesive layer thickness h tends to zero, then the elastic strain energy absorbed by the layer is negligible. A full analytical derivation was proposed [9] for a similar geometry of concentric tubes joined by an adhesive at their interface and subjected to axial load. The link between critical load for crack propagation, adhesion and geometry holds to be:

$$N_c = \sqrt{4\pi R G_a \frac{E_2 A_2}{E_1 A_1}(E_1 A_1 + E_2 A_2)} \quad (1)$$

where A_i are cross-section areas, E_i are Young's moduli and their product represent the axial rigidity of tubular elements in tension and the internal element have radius R (Fig. S3a). N_C is the critical load for which the critical strain energy release rate G_a is reached. Eq.1 is applied to bodies #1, #2 in Fig. S3a where the body #2 is cylindrical and it could be extended to a modified configuration shown in Fig. S3b that resembles to the CMFs structure of D, D+GO and D+SiC.

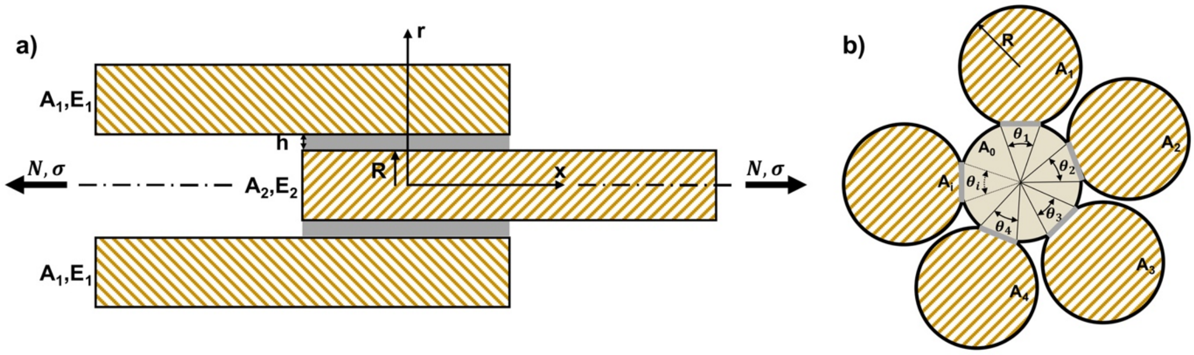

Figure S3. a) Depiction of tubular adhesive joint subjected to axial load, simplified for a tube-cylinder circular and co-axial contact mediated by an adhesive film, in grey. b) Schematic representation of the cross-section of previous joint for the case of several identical aligned microfibrils with partial adhesive contact described by θi. Ai are the cross-sections of the CMFs surrounding the central one, they have an Elastic Modulus of Ei. This configuration is depicting D, D+GO and D+SiC sample.

D, D+GO and D+SiC have equivalent cellulose microstructures (Fig. 2a), thus the only structural differences can be found in the interface layer, where nanoparticles are located. The differences between the concentric tubular configuration presented in [9] and our systems lies in the area of contact between different CMFs that is partial but have multiple areas of contact and does not extend to the entire external surface as for the tubes (Fig. S3a,b).

We propose to estimate if the nanoparticles at the CMF interfaces generate an increase in critical strain energy release at the microscale, which could cause the macroscopic enhancements in the stress of failure and toughness modulus in the D+GO and D+SiC samples (Fig. 3). Passing from a discrete analytical to a local model requires to substitute the axial force N with a constant distributed pressure σ on the x-axis that is generated as a consequence of the uniaxial tensile load conditions. The two models are linked if a catastrophic failure of the samples is considered. In particular, the bulk material exhibits a stable crack propagation when the applied load σ reaches a critical load σ_c, which is computed as the tensile strength of the mechanical tests.

Taking into account these considerations, Eq.1 can be rewritten to better suit the configuration of CMF in cellulose nanocomposites. The partial contact between CMFs is depicted in Fig. S3b where the central fibril is in contact with multiple fibrils, each for an arc of θ_iR. As the nanoparticles are filling vacancies inside the structure, the key in comparing different samples is that the contact area between microfibrils remains unchanged in D, D+GO, D+SiC, hence nanoparticles play a role only in the determination of fracture energy Ga. Comparing the results with the analytical case in [9], we can consider crack nucleation to be a statistical phenomenon since the problem is symmetrical. This scrutiny implies that two terms in the ratio between axial rigidities in Eq.1 can indifferently be inverted and thus the ratio can be considered equal to 1 for cellulose nanocomposites. Finally, the following correlation is obtained:

$$\sigma_c \propto \sqrt{\sum_i (\theta_i R) \cdot G_a \cdot \left[E_0 A_0 + \sum_i (E_i A_i)\right]} \quad (2)$$

Eq. 2 links the failure strength to the critical value of strain energy release rate (G_a), i.e. fracture energy of the interface, for which the crack propagates [8, 9]. E_i and E_0 are to be considered identical since the load bearing structure of the composite is the same, so they can be reduced to E and extracted from the summation. To compare different sample groups (D, D+GO, D+Si), a second index j is inserted to label the quantities dependent on the three groups.

$$\sigma_{c,j} \propto \sqrt{\sum_i (\theta_{i,j} R_j)} \cdot \sqrt{\sum_i A_{i,j}} \cdot \sqrt{E_j G_{a,j}} \quad (3)$$

The first two terms in the product in Eq.3 are identical under the hypothesis of identical configuration and microstructure, therefore the relation can be rewritten as follows:

$$\sigma_{c,j} \propto \sqrt{E_j G_{a,j}} \quad (4)$$

where the tensile strength σ_c is a function of E_j, $G_{a,j}$, which are sample-related values. E_j was computed as the elastic modulus measured upon tensile testing for each sample. Then, the average improvements in interfacial fracture energy for the two set of nanoparticles can be computed:

$$\frac{\bar{G}_{a,D+GO}}{\bar{G}_{a,D}} = \frac{\bar{\sigma}^2_{c,D+GO}}{\sigma^2_{c,D}} \cdot \frac{\bar{E}_D}{\bar{E}_{D+GO}} \quad (5a)$$

$$\frac{\bar{G}_{a,D+SiC}}{\bar{G}_{a,D}} = \frac{\bar{\sigma}^2_{c,D+SiC}}{\bar{\sigma}^2_{c,D}} \cdot \frac{\bar{E}_D}{\bar{E}_{D+SiC}} \quad (5b)$$

where the two ratios $G_{a,D+GO}/G_{a,D}$ and $G_{a,D+SiC}/G_{a,D}$ represent the average interfacial fracture energy improvements for the 2 sets of nanocomposite reed. They are linked with the average failure stress $\sigma_{c,j}$ and elastic modulus E_j of each set of samples. The results show that the intercalation of GO the interfacial fracture energy is improved by 28%, and by 47% after SiC intercalation.

Interfacial fracture energy improvements	
$\frac{\bar{G}_{a,D+GO}}{\bar{G}_{a,D}}$	**1.28±0.33**
$\frac{\bar{G}_{a,D+SiC}}{\bar{G}_{a,D}}$	**1.47±0.36**

Table S1. Interfacial fracture energy improvements for D+GO and D+SiC samples.

In summary, the estimation of the interfacial fracture energy improvements in Table S1 are in line with the results of the mechanical tests that show a higher tensile and flexural performance after the intercalation of SiC nanoparticles rather than GO. Finally, a linear correlation is obtained between elastic strain energy release rate and toughness modulus reported in Fig. 3f.

Note 4 - Video of the burning tests

Attached file: Video S1 - Burning tests.

List of Abbreviations

GO graphene oxide; SiC silicon carbide; R giant reed; TC thermo-compressed; D densified; D+GO densified + graphene oxide; D+SiC densified + silicon carbide; CMF cellulose microfibril; XPS X-ray photoelectron spectroscopy; DSC differential scanning calorimetry; TGA thermogravimetric analysis; EDX energy dispersive X-ray; SEM scanning electron microscope

References

1. Gargulak, J. D.; Lebo, S. E.; McNally, T. J., Lignin. *Kirk-Othmer encyclopedia of chemical technology* **2001**.
2. Beamson, G.; Briggs, D., High resolution XPS of organic polymers: The Scienta ESCA 300 database. *Surface and Interface Analysis* **1992,** *20* (3), 267-267.
3. Goda, K.; Sreekala, M.; Gomes, A.; Kaji, T.; Ohgi, J., Improvement of plant based natural fibers for toughening green composites—Effect of load application during mercerization of ramie fibers. *Composites Part A: Applied science and manufacturing* **2006,** *37* (12), 2213-2220.
4. Li, X.; Tabil, L. G.; Panigrahi, S., Chemical treatments of natural fiber for use in natural fiber-reinforced composites: a review. *Journal of Polymers and the Environment* **2007,** *15* (1), 25-33.

5. Li, Y.; Ruan, R.; Chen, P. L.; Liu, Z.; Pan, X.; Lin, X.; Liu, Y.; Mok, C.; Yang, T., Enzymatic hydrolysis of corn stover pretreated by combined dilute alkaline treatment and homogenization. *Transactions of the ASAE* **2004,** *47* (3), 821.
6. Fratzl, P.; Burgert, I.; Gupta, H. S., On the role of interface polymers for the mechanics of natural polymeric composites. *Physical Chemistry Chemical Physics* **2004,** *6* (24), 5575-5579.
7. Gent, A.; Yeoh, O., Failure loads for model adhesive joints subjected to tension, compression or torsion. *Journal of Materials Science* **1982,** *17* (6), 1713-1722.
8. Pugno, N.; Carpinteri, A., Strength, stability and size effects in the brittle behaviour of bonded joints under torsion: theory and experimental assessment. *Fatigue & Fracture of Engineering Materials & Structures* **2002,** *25* (1), 55-62.
9. Pugno, N.; Carpinteri, A., Tubular adhesive joints under axial load. *Journal of applied mechanics* **2003,** *70* (6), 832-839.
